# Resveratrol mediates its anti-cancer effects by Nrf2 signaling pathway activation

**DOI:** 10.1186/s12935-021-02280-5

**Published:** 2021-10-30

**Authors:** Matin Alavi, Tahereh Farkhondeh, Michael Aschner, Saeed Samarghandian

**Affiliations:** 1 Faculty of Medicine, Mashhad University of Medical Sciences, Mashhad, Iran; 2 Cardiovascular Diseases Research Center, Birjand University of Medical Sciences, Birjand, Iran; 3 Faculty of Pharmacy, Birjand University of Medical Sciences, Birjand, Iran; 4 Department of Molecular Pharmacology, Albert Einstein College of Medicine, Forchheimer 209, 1300 Morris Park Avenue, Bronx, NY 10461 USA; 5 Noncommunicable Diseases Research Center, Neyshabur University of Medical Sciences, Neyshabur, Iran

**Keywords:** Resveratrol, Cancer, Nrf2

## Abstract

**Aim and background:**

Cancer represents a major health problem with an exceedingly high toll on the patients, their families, and the economy. Cancers are also associated with high mortality rates. Existing therapies for cancer are generally ineffective with many side effects.

**Method:**

A search was conducted on Pubmed, Google Scholar, Scopus, and web of science databases, and articles related to anticancer effects of resveratrol were collected.

**Results:**

Resveratrol is a natural compound that can activate the Nrf2 transcription factor. Nfr2 translocates to the nucleus and induces antioxidant gene expression. In different cell lines, resveratrol can increase apoptosis and inhibit the proliferation of cancer cells.

**Conclusion:**

We found that resveratrol shows efficacy for the treatment of cancer, but due to high controversy on the Nrf2 signaling pathway and mechanisms of resveratrol action, additional studies should be conducted to better characterize its mode-of-action in cancer.

**Graphical Abstract:**

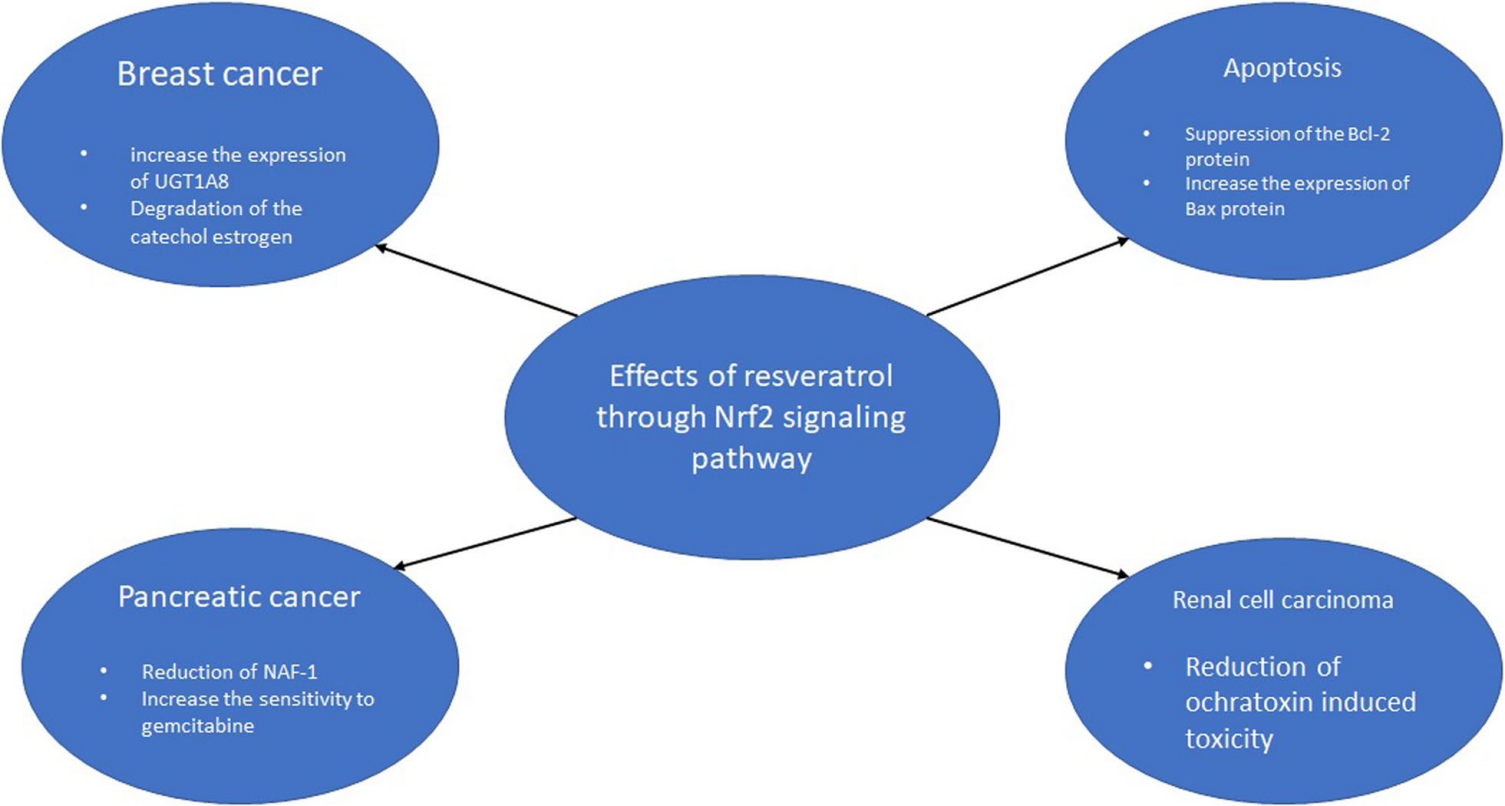

## Introduction

Resveratrol belongs to the flavonoids group and can be found in various fruits (e.g. berries, grapes, red wine, and peanuts). In addition to its anticancer effects, it has anti-diabetic, antioxidant, anti-inflammatory effects. Resveratrol has beneficial effects against drug resistance in cancer and can increase the sensitivity of cells to chemotherapeutic drugs. Resveratrol also has protective properties on the liver, heart, and brain [1]. Cancer and cardiovascular disease are two major health problems with high mortality rates [2]. Cancer is responsible for many deaths that occur in a year. In 2020, it is estimated that about 10 million cancer death has occurred and 19.3 million new cases were diagnosed with cancer [3]. Existing therapies for cancer are generally not effective. Current therapies for cancer including chemotherapy and radiotherapy have multiple side effects and resistance to them may develop over time. Chemotherapeutic drugs simultaneously affect both normal and cancer cells. Cancer patients treated with chemotherapy lose their hair and their bone marrow is damaged which may lead to aplastic anemia [4]. Patients who are treated with radiotherapy display many side effects including lymphopenia, thrombocytopenia, and neutropenia [5–7]. Radiotherapy damage to stem cells of bone marrow may be teratogenic for the fetus [8, 9]. It also affects the skin and causes radiodermatitis and increases the risk of secondary cancer following therapy [10, 11]. In addition, radiotherapy damages the DNA and causes apoptosis and cell death [12]. Thus, discovering new cancer therapeutic approaches is necessary and timely. Resveratrol is a natural product and has anticancer effects. It can activate the Nrf2 signaling pathway and reduce oxidative stress. In this review, we summarized the anticancer effects of resveratrol which are mediated via the activation of the Nrf2 signaling pathway.

## Oxidative stress and cancer pathogenesis

**Fig. 1:**
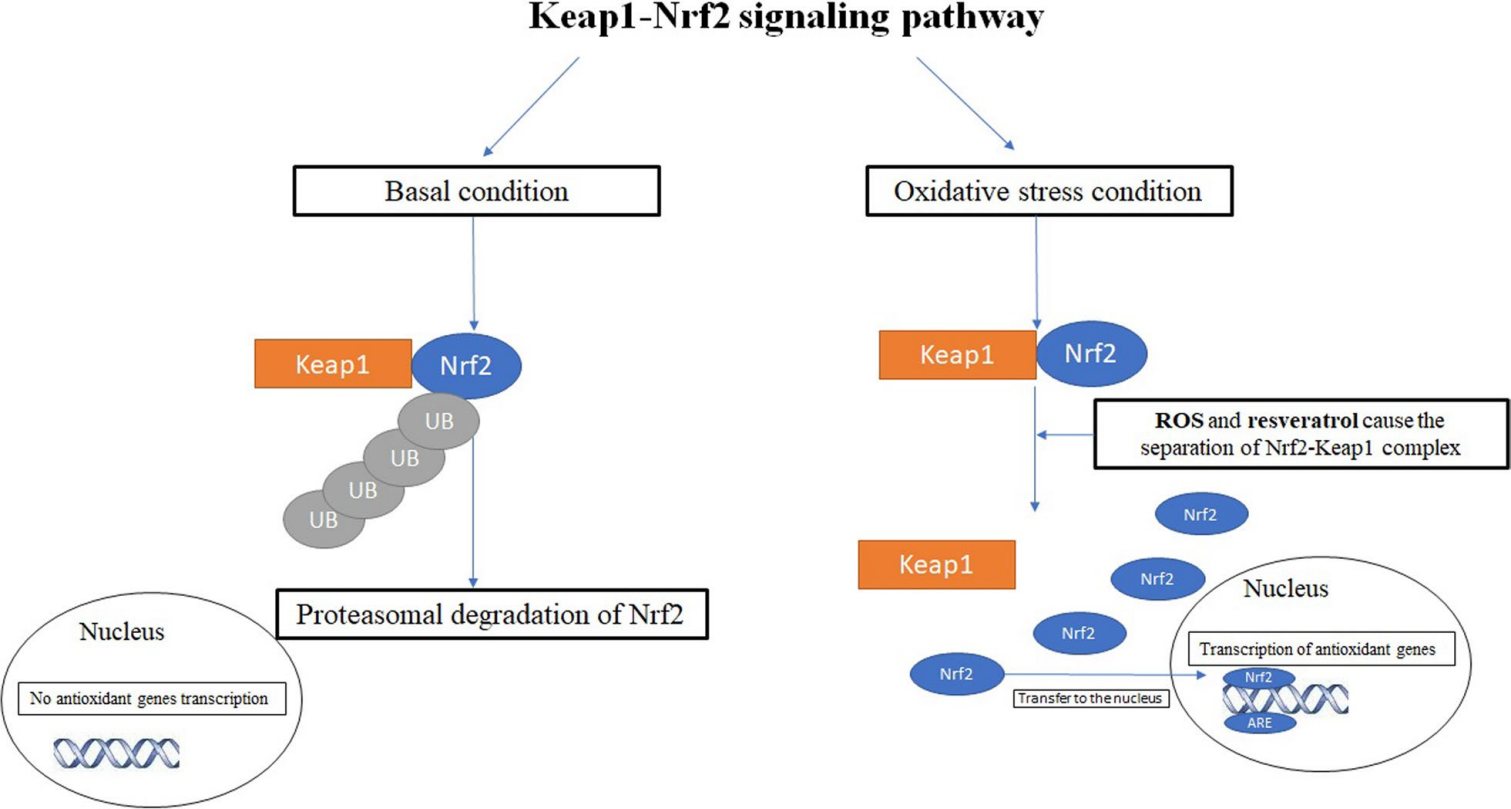
The keap1-Nrf2 pathway

Oxidative stress is a trigger and occurs in many diseases, such as diabetes, cancers, and neurological disorders. Various metabolic pathways lead to the production of reactive oxygen species, referred to as ROS (e.g. O_2_–, H_2_O_2_, OH–, O_3_). For instance, UV radiation, enzymes such as NADPH oxidase, and chemical substances, such as alcohol can produce oxidative stress in cells. Cells also possess antioxidant enzymes (e.g. catalase and superoxide dismutase) that can reduce ROS and decrease restore their redox status. HO-1 (heme oxygenase-1) is an antioxidant enzyme and its levels increase upon resveratrol treatment [13–15]. In normal cells, low levels of ROS have been implicated in signal transduction, phagocytosis, inflammation, and activation of enzymes [4, 15]. In turn, ROS production in tumor cells is elevated cells as a consequence of increased metabolic rate, gene mutation, and relative hypoxia, and excess ROS are quenched by increased antioxidant enzymatic and non-enzymatic pathways in the same cells. Furthermore, ROS activates signaling pathways related to the metastasis of tumors. ROS can induce apoptosis by activation of caspase enzymes and several antioxidant substances prevent cells from undergoing this process [15]. In addition, oxidative conditions in cancer cells increase VEGF levels for angiogenesis. In cancer therapy, there are anti-angiogenesis antibodies that can block the VEGF receptor [16] (Fig. 1).

## Nrf2 signaling pathway

Cap’n’collar (Cnc) transcription factors family has several members including Nrf2, Nrf3, Nrf1. Nrf2 (nuclear erythroid-2 related factor 2) is a transcription factor that can stimulate the antioxidant enzymes. It can regulate the oxidative stress of cells by activating the genes which are related to cellular stress [1, 17]. Nrf2 has tumor suppressor effects and also can increase the proliferation in cancer cells. It has been shown that in many cancers the expression level of Nrf2 is elevated [18]. In addition, in cancer cells, the overexpression of Nrf2 leads to resistance to chemotherapy and radiotherapy. Nrf2 has seven domains (Neh1 to Neh7) and two binding sites (ETGE and DLG). The most important domain for Nrf2 is Neh2 that has seven lysin amino acids [18]. The activation of the Nrf2 transcription factor due to its antioxidant properties may be effective in cancer therapy. But there is a controversy on whether activation of Nrf2 is of clinical benefit in cancer therapy or is a carcinogen? Nrf2 has been referred to as a double-edged sword. In addition to its cytoprotective and chemoprevention effects, the activation of Nrf2 results in inhibition of apoptosis, induction of proliferation, and also enhancement of cell survival [19]. During chemotherapy, antioxidant levels of β-carotene, vitamin C, and E are decreased. In addition, chemotherapeutic and antineoplastic drugs (e.g. daunorubicin, and epirubicin) can increase ROS levels and induce oxidative stress and attenuate cancer cells death. It has also been suggested that reduction in oxidative conditions in cancer cells may enhance the anticancer effects of antineoplastic drugs [4]. In a study by DeNicola et al., it was shown that Oncogenes including Kras, Myc, and Braf genes suppressed ROS production and increased the transcription of Nrf2 in cells [20] (Table 1).

**Table 1:** Effects of resveratrol on cancer cell lines

Cell line/method	Signaling pathway	Mechanism	Results	Refs.
MCF-10A/Res	NRF2–UGT1A8 pathway	Overexpression of NRF2 and UGT1A8	Resveratrol can regulate the estrogen level in cells	Zhou et al. [28]
MCF-10A/Res	NRF2-mediated protective pathways		Res can induce apoptosis and increase the Nrf2 and inhibit the E2-induced breast cancer	Singh et al. [35]
MCF-10F/Res	induction of Nrf2 transformation into the nucleus through increasing NQO1 expression. (Nrf2-Keap1-ARE pathway)	increase and decrease the expression of NQO1 and CYP, respectively	Resveratrol is a chemopreventive for breast cancer and decreases estrogen metabolism	Fang et al. [36]
MCF10A/Res	Nrf2 and BCR1	Overexpression of Nrf2 can reduce oxidative stress and prevent the tumorigenesis of the BCR1 gene	Resveratrol and sulforaphane decrease the ROS accumulation via activation of Nrf2 expression	Hyo et al. [37]
MCF-10A, MCF-10F/ HPIMBD, TIMBD		increase the expression of NQO1 and SOD3 and activation of Nrf2	HPIMBD and TIMBD can prevent breast cancer due to their antioxidant properties	Anwesha et al. [29]
RA-FLS/Res	Nrf2-Keap1	Overexpression of Nrf2 and reduction in the expression of Keap1, reduction in the ROS and MDA production	Induction of apoptosis through Bcl2, inhibition of cell migration and proliferation, restoring the ROS accumulation caused by H_2_O_2_	Zhang et al. [30]
A549/Res	Nrf2-Keap1	Res loaded in nanoparticles compared to Res showed the better effects in the elevation of Nrf2 level	Activation of Nrf2-Keap1 pathway, restoring the ROS accumulation caused by H_2_O_2_	Kim et al. [20]
Animal study in mice/Res	Nrf2/HO-1 pathway	Suppress the IL-8, NF-κB, and iNOS expression and also increase in the HO-1 and Nrf2	Res can activate the Nrf2/HO-1 pathway	Zhang et al. [38]
An animal study in rats with oxidative stress damage in hepatocytes/Res		Increase in the Nrf2 mRNA concentration and antioxidant enzymes (e.g. catalase, glutathione peroxidase, and also superoxide dismutase	Res can protect the hepatocytes in oxidative stress conditions	Rubiolo et al. [39]
HL-60/ effects of Res in a leukemia cell line that is resistant to ADR	PI3K/Akt/Nrf2 Pathway	PI3K/Akt/Nrf2 expression are decreased in cell line	Res showed the antiproliferative activity against the cell line and promote the inhibition of cells by ADR	Yongjun et al. [25]
Human keratinocytes/Res	Nrf2–KEAP1 pathway	Activation of antioxidant in the skin, Increase in the glutathione peroxidase-2, glutamylcysteinyl ligase, and GSH	Res protects the skin cells from oxidative stress	Soeur et al. [32]
A375SM/Res		Increase in the p27, and p21 gene expression and reduction of Bcl-2, Nrf2, and cyclin B expression	Res can induce cell cycle arrest and apoptosis and inhibit the cell's proliferation but in contrast, it elevates ROS production and oxidative stress	HEO et al. [33]
Animal study in male Wistar rats with renal carcinoma/ Res + sitagliptin	Nrf2/HO-1 pathway, STAT3/NF-κB signaling	Reduction in the TNF-α, STAT3, LDH, TGF-β1, and IL-6. Res suppress the expression of P-glycoprotein	sitagliptin and Res improve kidney function in rats. They suppress inflammation and oxidative stress and have better effects when they are used together	Kabel et al. [40]
HEK293/Res		Increase in the repair enzyme of DNA, OGG1, GSH, and decrease in the expression of Nrf2	Res has antioxidant and anticancer effects and Increases repair enzymes of DNA, Res can remove the toxicity of OTA	Raghubeer et al. [34]
Pancreatic cancer cell line/Res	ROS/Nrf2 Pathways	Downregulation of NAF-1, induction of ROS accumulation, activation of Nrf2	Res can inhibit the proliferation and induce apoptosis in cancer cells	Cheng et al. [13]
MSTO-211H/combination of Res with clofarabine	PI3K/Akt signaling	Inactivation of Nrf2, suppression of HO-1	High Inhibitory effects on growth cells when they were used together	Lee et al. [31]
oogonial stem cell/Res		Activation of Nrf2	Res restore the oxidative stress and by chemotherapy in oogonial stem cells. it can reduce the H_2_O_2_ and cytotoxicity effects of chemotherapy	Meng et al. [15]

*Res* Resveratrol, *E2* 17β-estradiol, *UGT1A8* UDP-glucuronosyltransferase 1A8, *AhR* hydrocarbon receptor, *CYP* cytochrome P450, *NQO1* NAD(P)H quinone oxidoreductase 1, *SOD3* superoxide dismutase 3, *RA-FLS* rheumatoid arthritis fibroblast-like synoviocyte, *MDA *malondialdehyde, *HO-1* Heme Oxygenase-1, *ADR* adriamycin, *TIMBD* 4-(E)-(*p*-tolylimino)-methylbenzene-1,2-diol, *HPIMBD* 4-(E)-(4-hydroxyphenylimino)-methylbenzene, 1, 2-diol, *OGG1* oxoguanine glycosylase 1, *GSH* glutathione, *OTA* ochratoxin A, *NAF-1* nutrient-deprivation autophagy factor-1, *HO-1* heme oxygenase-1

## The keap1-Nrf2 pathway

The Keap1-Nrf2 signaling pathway is essential for the regulation of oxidative stress [19, 21]. In the basal condition, Nrf2 and keap1 are connected and whenever cells are placed in oxidative condition, Nrf2 is separated from Keap1, transfer to the nucleus, and activate the antioxidant genes [19].

Keap1 has three domains that can bind to ETGE and DLF motifs from the Nrf2 protein. Keap1 and Nrf2 form a complex with Cullin3 and E3 ubiquitin ligase. The oxidation of cysteine sulfhydryl groups in the oxidative stress condition causes the separation of Nrf2 from Keap-1. Then Next, Nrf2 translocates to the nucleus forming a heterodimer complex with Maf (musculoaponeurotic fibrosarcoma) and binds to an ARE (antioxidant response element) enhancer [18, 22].

Nrf2 binds to the NF-E2 site of the β-globin gene. This molecule has cytoprotective and chemoprevention activity [23]. Several substances induce Nrf2 activation (e.g. Hydrogen sulfide, nitrogen oxide, physical activity, lipid peroxidation, and curcumin). Keap1 (formerly known as an Nrf2 inducer) is a protein that the stress molecule can bind to the cysteine amino acid. Indeed, Keap1 protein is a negative controller of the Nrf2. Keap1 has oxidative sensors and can detect oxidative stress such as ROS in the cells [23, 24]. The Keap1-Nrf2 pathway regulates the anabolic pathways in the cells that are necessary for the reduction of oxidant (for example, NADPH that is produced in the pentose phosphate pathway) [24, 25].

## PI3K/AKT pathway

The class I_A_ of the PI3K family has been shown to be responsible for cancer progression. It (class I_A_) has two subunits (p85 and p110 subunits). The PIP2 (phosphatidylinositol-4,5-bisphosphate) is the substrate of PI3K. when growth factors bind to their receptors on the surface of cells, the inhibitory effect of the p85 subunit dissociates from the p110 subunit. In addition, the p110 subunit can be activated by ROS. The phosphorylation of PIP2 by p110 results in PIP3 (phosphatidylinositol-3,4,5-trisphosphate) production. Next, PIP3 binds to PDK1 and AKT proteins, leading to phosphorylation of AKT protein by PDK1 and activation of numerous enzymes. AKT can phosphorylate transcription factors and proteins involved in cell survival [26]. In a leukemia cell line activation of the PI3K/AKT pathway has been shown to increase Nrf2 expression [27].

## Effects of resveratrol on various types of cancers

Estrogen is a steroid hormone that increases the risk of breast cancer. Due to the reduction of estrogen in menopausal women, the risk of osteoporosis and cardiovascular disease is increased in this group. As a treatment for this condition, estrogen as a hormone therapy has been administrated to menopausal women. Yet, estrogen is a carcinogen, and can significantly increase the risk of breast cancer [28, 29]. Thus, the regulation of estrogen levels is important in the prevention of breast cancer.

Catechol estrogen is a carcinogen for breast cancer. UGT1A8 is an enzyme that can metabolize the catechol estrogen. Resveratrol can increase the expression of UGT1A8 through activation of the Nrf2 gene expression and degrade the catechol estrogen. Indeed, Nrf2 affects the promoter of the UGT1A8 gene and induces UGT1A8 gene activation [30]. Anwesha et al. synthesized two analogs of resveratrol (HPIMBD and TIMBD) and compared their antioxidant and cytotoxicity effects in the presence or absence of resveratrol. They reported that these analogs do not have antiproliferative or cytotoxicity effects against the MCF-10A cell line. But, compared to resveratrol can more efficaciously induce Nrf2 expression. HPIMBD and TIMBD also increased SOD3 enzyme expression which is responsible for the detoxification of ROS, significantly attenuating ROS generation in this cell line [31].

As noted above, oxidative stress may trigger carcinogenesis and increase cell proliferation [32]. By activating the Nrf2 signaling pathway resveratrol protects cells from oxidative stress-induced damage. Zhang et al. found that resveratrol can increase the Nrf2 and HO-1 expression and in contrast, it reduces the ROS production and Keap1 expression. When treated with resveratrol, cell proliferation was inhibited and apoptosis was induced secondary to suppression of the Bcl-2 protein and increased expression of Bax protein [32].

Cheng et al. showed that resveratrol can induce apoptosis and inhibit cell proliferation. Resveratrol activated the Nrf2 through ROS production [13]. Lee et al. used the combination of resveratrol and clofarabine on the MSTO-211H cell line. When combined, their inhibitory effects against cell growth were promoted. Reduction in Nrf2 protein expression levels and increased cell viability were reported in cells are treated with the combination of resveratrol and clofarabine [33].

Soeur et al. used keratinocytes to investigate the antioxidant properties of resveratrol in skin cells, showing the latter can increase the antioxidant enzymes, such as glutathione peroxidase-2 by activating the Nrf2-Keap1 pathway [34]. Reduction in Nrf2 expression by resveratrol was also reported. HEO et al. showed the antiproliferative effects of resveratrol against malignant melanoma cells, reporting that resveratrol induced apoptosis by increasing Bcl-2 expression levels, but decreased Nrf2 expression level in melanoma cells [35]. In a leukemia cell line, aberrant activation of the PI3K/AKT/Nrf2 pathway inhibited apoptosis and increased cell proliferation [27].

The effects of resveratrol in pancreatic and renal cell carcinoma also have been investigated in vitro. Shanel et al. reported the ameliorative activity of resveratrol against toxicity induced by ochratoxin in human embryonic kidney cells (HEK293 cell). Some fungi such as *Penicillium* and *Aspergillus* can produce it. Ochratoxin can induce oxidative stress. It has nephrotoxin activity and causes renal dysfunction. Results showed that after 48 h resveratrol elevate the expression of Nrf2 cells. in conclusion, resveratrol can be regarded as a good choice for Ochratoxin-induced toxicity and has chemo-preventive properties [36].

Resveratrol increases the pancreatic cancer cells' sensitivity to gemcitabine by its effect on NAF-1 (nutrient-deprivation autophagy factor-1) and Nrf2 signaling. Liang et al. showed that Resveratrol can activate the Nrf2 signaling and reduce the expression of NAF-1 that has anti-apoptotic activity. in addition to induction of apoptosis, resveratrol showed the antiproliferative activity against pancreatic cancer cells. in conclusion, new drugs for reducing the transcription or activity of NAF-1 (e.g. resveratrol) may be effective in the treatment of pancreatic cancer [13].

## Resveratrol effect on tumor microenvironment

Resveratrol can regulate the tumor microenvironment via modulating oxidative stress, angiogenesis, fibrosis, and the immune system [37]. In the tumor cell microenvironment, ROS levels increase and lead to apoptosis by activating p53. It was found that resveratrol has two contradictory impacts on oxidative stress. In its therapeutic effect, it elevates oxidative stress to prevent cancer cell progression [38]. In its chemopreventive effect, it can act as a ROS scavenger to sustain cells from mutations. Resveratrol can affect different innate immune cells which are involved in the regulation of tumor microenvironment. It was found that resveratrol inhibited the activation of M2 macrophage and also induced repolarization of tumor-associated macrophages (TAM) from M2 to M1, resulted in tumor suppression and metastasis. M1 is the active form of macrophage in normal cells that produces several cytokines. In the tumor microenvironment condition, reprogramming M2 toward the M1 phenotype is associated with the overproduction of inflammatory cytokines leading to cell destruction [39]. Resveratrol can also decrease immune tolerance in tumor cells by inhibiting the enzyme indoleamine 2,3-dioxygenase (IDO) expression and activity in dendritic cells resulted in regulation of cytotoxic T cell polarization to increase its antitumor effect. Treatments with anti-angiogenesis agents have been focused on as a suitable strategy among patients with solid tumors to prevent tumor progression. Resveratrol was effective on angiogenesis through an inhibitory direct effect on vascular endothelial growth factor (VEGF) generation and also inhibiting the hypoxia-inducible factor (HIF)-1generation and leads to preventing VEGF secretion [40]. Fibroblasts are involved in the tumor’s progression by producing platelet-derived growth factor (PDGF), stromal cell-derived factor 1 (SDF1), VEGF, and basic fibroblast growth factor (bFGF). Resveratrol can inhibit the tumor cell viability by decreasing several fibrogenic mediators including a-SMA, type I collagen, and fibronectin [40].

## Clinical trial studies related to anti-tumor effects of resveratrol

There are few clinical studies related to the anti-cancer activity of Res. A clinical trial conducted on the protective impact of plant-based Res on colon cancer patients showed that this agent could not inhibit the expression of Wnt, myc, and cyclin D1genes in a sample of patients [41]. Patel et al. reported that resveratrol and its metabolites (resveratrol-3-*O*-glucuronide, resveratrol-4′-*O*-glucuronide, resveratrol-3-*O*-sulfate, resveratrol-4′-*O*-sulfate, resveratrol sulfate glucuronide and resveratrol disulfate) were present in the operated colorectal tissue [42]. Howells et al. reported higher levels of resveratrol in plasma and hepatic tissues after SRT501administration in patients with colorectal cancer and hepatic metastasis who were scheduled to undergo hepatectomy [43]. No significant alteration was observed in AKT1, GSK-3, survivin, and PARP biomarkers [43]. Zhu et al. evaluated the resveratrol impact on the methylation of certain proteins in women with breast cancer. Sample biopsy demonstrated invasive breast cancer with atypical hyperplasia [44]. It was found that 5 or 50 mg/2 per day of trans-resveratrol for 12 weeks reduced methylation of RASSF-1a, leading to a decrease in prostaglandin E2 (PGE2) expression in breast cancer [45]. Brown et al. [46] reported that administration of 4000 mg/patient of Res was safe among patients with recurring prostate cancer [47]. Another trial indicated use of two doses of resveratrol for 4 months decreased blood androstenedione, dehydroepiandrosterone, and dehydroepiandrosterone-sulfate concentrations without change in the size of the prostate among patients with benign prostate hyperplasia [48]. However, there are some reports related to the side effects of Res in cancer patients. It was indicated that administration of Res (5 mg/day for 6 days) increased protein carbonyl levels in patients with colorectal cancer [49]. SRT501 supplements daily caused kidney toxicity in patients with multiple myeloma at the second phase of the clinical trial, led to a patient’s death [50]).

## Conclusion and future perspectives

The evidence from experimental studies suggests that resveratrol has a protective effect against several cancers by inhibiting the expression and levels of Nrf2 in cancerous samples. In addition, it can induce apoptosis and inhibit cell proliferation. Resveratrol may be effective in combination with other chemotherapeutics agents. Although, most of the studies indicated the safety of Resveratrol; however, there are some reports related to its toxicity due to dosing regimen. Current data related to trials on the effectiveness of resveratrol in patients with a different type of cancer treatment are still very few. In addition, the studies have a low sample size. The molecular mechanisms involved in the protective effects of Res against cancer were not evaluated in human samples. Therefore, more clinical trials are needed to find the exact doses and duration for cancer treatment and prevention and also determine molecular targets triggered by Res. In addition, a novel formulation of Res with nano delivery systems should be designed and evaluated their pharmacokinetic and pharmacodynamics in cancer patients.

## Data Availability

All data are available in the manuscript.
